# Corallith bed of the endangered coral *Cladocora caespitosa* in the South Adriatic Sea

**DOI:** 10.1038/s41598-025-01554-6

**Published:** 2025-05-14

**Authors:** Giovanni Chimienti, Andrea Tursi, Alessia Logrieco, Silvia Notarangelo, Francesco Mastrototaro

**Affiliations:** 1 Department of Biosciences, Biotechnology, and Environment, University of Bari Aldo Moro, Via Orabona 4, 70126 Bari, Italy; 2 CoNISMa, National Interuniversity Consortium for Marine Sciences, Piazzale Flaminio 9, 00197 Rome, Italy; 3 Department of Earth and geo-environmental sciences, University of Bari Aldo Moro, Via Orabona 4, 70126 Bari, Italy

**Keywords:** Habitat, Corallith bed, Population size structure, Conservation, Marine protected area, Tremiti Islands, Mediterranean sea, Ecosystem ecology, Ecosystem ecology

## Abstract

The colonial coral *Cladocora caespitosa* is a Mediterranean-endemic scleractinian structuring ecosystems of great conservation and ecological importance. Their nomenclature is here revised distinguishing between three different *C. caespitosa* formations: beds, grounds and reefs. Here we describe an extensive *C. caespitosa* bed at Tremiti Islands (Italy, Adriatic Sea), where thousands of colonies develop as free-living coral nodules (coralliths) on a soft, detritic bottom. Colonies were seldom present shallower than 15 m depth as well as on rocky bottoms, while their density increased with depth up to 700 colonies 100 m^− 2^ at 22 m. Their bathymetric occurrence was primarily influenced by seawater temperatures, particularly during summer, when a marked thermocline act as protection against high temperatures. The conservation status of the *C. caespitosa* bed was overall good, with low epibiosis/necrosis, and negligible bleaching. Demographic traits and size-structure of both colonies and polyps (through corallites) are reported. These rare coral formations might be affected by anthropogenic threats at both local and global scale, thus appropriate conservation measures are needed to avoid significant impacts in the future.

**Supplementary Information:**

The online version contains supplementary material available at 10.1038/s41598-025-01554-6.

## Introduction

The coral *Cladocora caespitosa* (Linnaeus, 1767) is a zooxanthellate scleractinian able to form extensive biogenic habitats in the Mediterranean Sea, where it is endemic. Thanks to its aragonitic skeleton, this long-living ecosystem-engineering species forms durable bioconstructions based on its round and cushion-shaped colonies. This coral can grow up to 6.2 mm year^− 1^^1^ depositing 1.1–1.7 kg CaCO_3_ m^− 2^ year^− 1^^2^, comparably to many tropical reef corals^3^. Thanks to its high plasticity^4^, *C. caespitosa* is known to constitute two distinct ecosystem types called beds and banks^5,6^. Beds are composed of numerous subspherical colonies, often 10–30 cm in diameter, forming dense populations with some space between colonies^6,7^. Banks are made up of large colonies, in contact with each other or with little space between them, forming large carbonate frameworks and covering several square meters in surface area^7,8^. Banks may originate from beds under conditions of undisturbed accretion, and mixed distributions of beds and banks can also occur^9,10^. Populations of *C. caespitosa* have been found mostly on rocky bottoms, although they can also occupy soft bottoms, including detritic seabed and rhodolith beds, where they can live as free-living coral nodules called coralliths^11^. Although currently found only in S. Espardelló islet (Formentera, Balearic Sea)^11,12^ and at Mljet National Park (Croatia, Adriatic Sea)^13^, it has been hypothesized that coralliths occurrence indicates a different *C. caespitosa* formation compared to former beds and banks^10^. In fact, different scleractinian species are known to grow as coralliths under specific conditions in the Atlantic, Indian, and Pacific oceans^14–17^. Similar bionodules or macroids are called rhodoliths when formed by calcareous rhodophytes^18–20^, and bryoliths when formed by bryozoans^21,22^. They can form beds when abundant, the most studied worldwide being rhodolith beds^23^.

Mediterranean fossil records show that *C. caespitosa* banks were more common during both the Pliocene^24^ and the Pleistocene^25^ than in the present time. Current populations are widespread throughout the Mediterranean Sea, but they mostly consist of small and sporadic colonies, while beds and banks have become rare^7,9,26^. The increase in seawater temperature and the recurrent marine heatwaves have been leading to an alarming deterioration of the conservation status of *C. caespitosa*, with several bleaching episodes and at least five major mortality events recorded in the last fifteen years^27–31^. Besides global warming, *C. caespitosa* is also vulnerable to a variety of anthropogenic pressures including the spread of alien species^32^, eutrophication^33^, pollution and litter^34,35^, as well as high sedimentation rates and coastal development^35^. Due to the multiple impacts affecting *C. caespitosa* and considering both the general decline and the poor capacity to recover after disturbances^9^, the species has been listed as endangered by the International Union for the Conservation of Nature (IUCN)^36^. Thus, understanding the bio-ecological traits of *C. caespitosa* populations—including distribution, demographic data, connectivity, conservation status and local threats—is essential to develop both appropriate conservation actions and sustainable management of human activities.

In this study, we report the finding of an extensive *C. caespitosa* population on the soft, detritic bottoms of the Tremiti Islands Marine Protected Area (MPA; Italy, Southern Adriatic Sea). Given the dominant presence of corallith growth forms, we describe a corallith bed and we propose to revise the nomenclature of the *C. caespitosa* formations distinguishing between reefs (previously known as banks), grounds (previously known as beds) and corallith beds. Density, cover, morphometry, size-frequency distribution, and conservation status were assessed on more than 5000 corallith colonies of *C. caespitosa* in three different study sites, from 14.5 to 22.0 m depth, and coupled with oceanographic data. Colonies aggregated in large beds, particularly below 17 m depth, where they seem to escape from extreme summer conditions, being able to reach high densities and cover.

## Results

### Corallith bed description and main bathymetric pattern

**Fig. 1:**
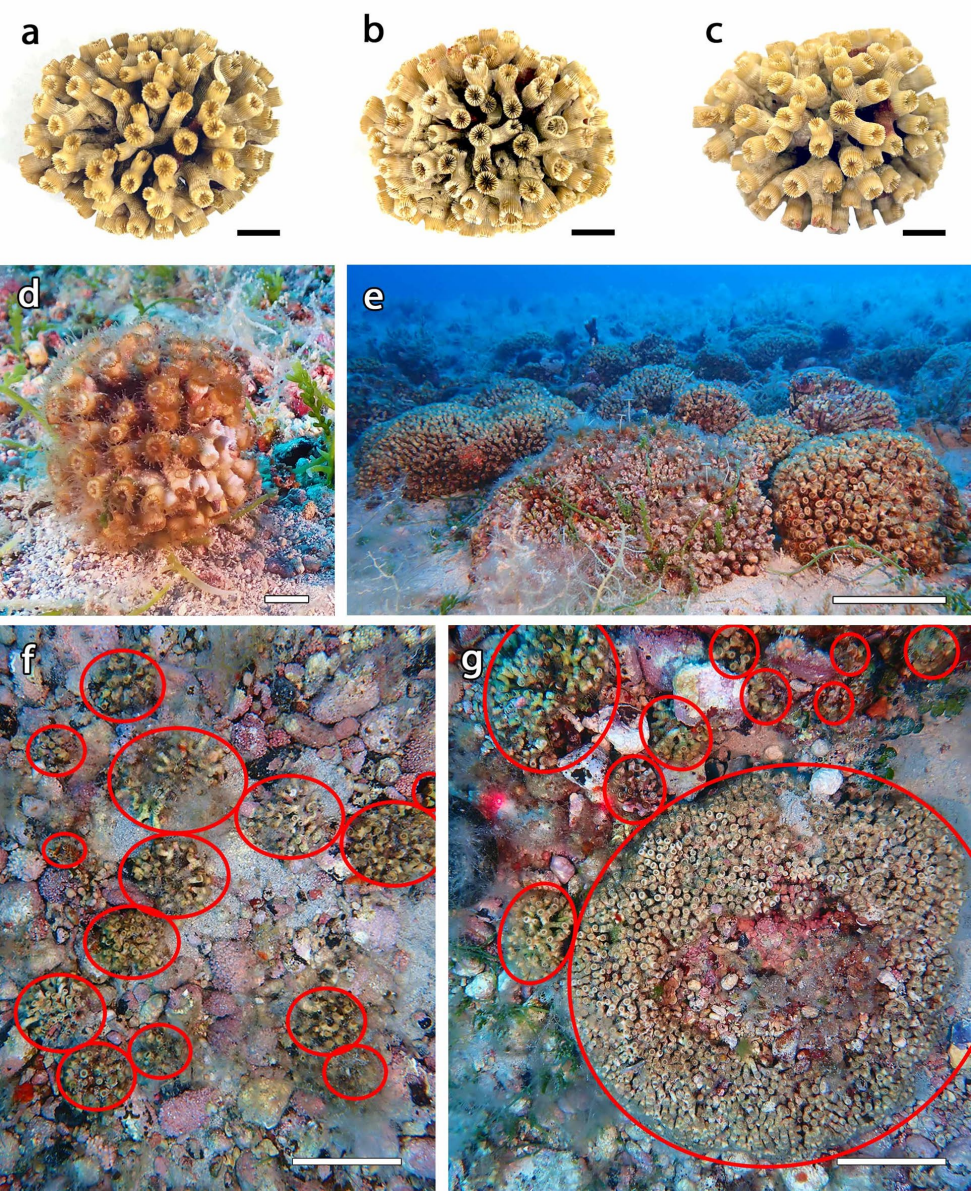
*Cladocora caespitosa* corallith bed of Tremiti Islands. Corallith sample in (**a**) dorsal, (**b**) ventral, and (**c**) lateral view; (**d**) corallith in situ; (**e**) bed with large coralliths; (**f**) example of small coralliths (red circles) with rhodoliths; (**g**) example of 9 small and 1 large corallith. Scale bars: a-d 1 cm; e-g 10 cm.

*Cladocora caespitosa* was found mainly on detritic bottoms, in the form of coralliths, i.e., unattached coral nodules with polyps growing in all directions (Fig. 1a–c). Most of the colonies were relatively small (< 10 cm) and spherical, while the largest ones tended to have irregular shapes and sometimes to fragment, all of them resulting unattached to the seabed. The lower part of the colonies, in contact with the seabed, showed dead polyps suggesting that these colonies developed without the need to adhere to a larger substrate (Fig. 1b). Smaller colonies were spherical with polyps in upright positions throughout the surface of the colony (Fig. 1d), providing evidence that they might roll during the initial phase of the colony development, until they became large and heavy enough not to be pushed by bottom currents (Fig. 1e), showing an effective adaptation to live on soft bottoms. A *C. caespitosa* corallith bed, intended as a large aggregation of coralliths, was found extending over an area of 11.6 hectares between 15 and 25 m depth, on a pebbly substrate with rhodoliths (Fig. 1f–g). Furthermore, *C. caespitosa* was just occasionally present on the rocky substrates in the same area, with a few, isolated colonies.

**Fig. 2:**
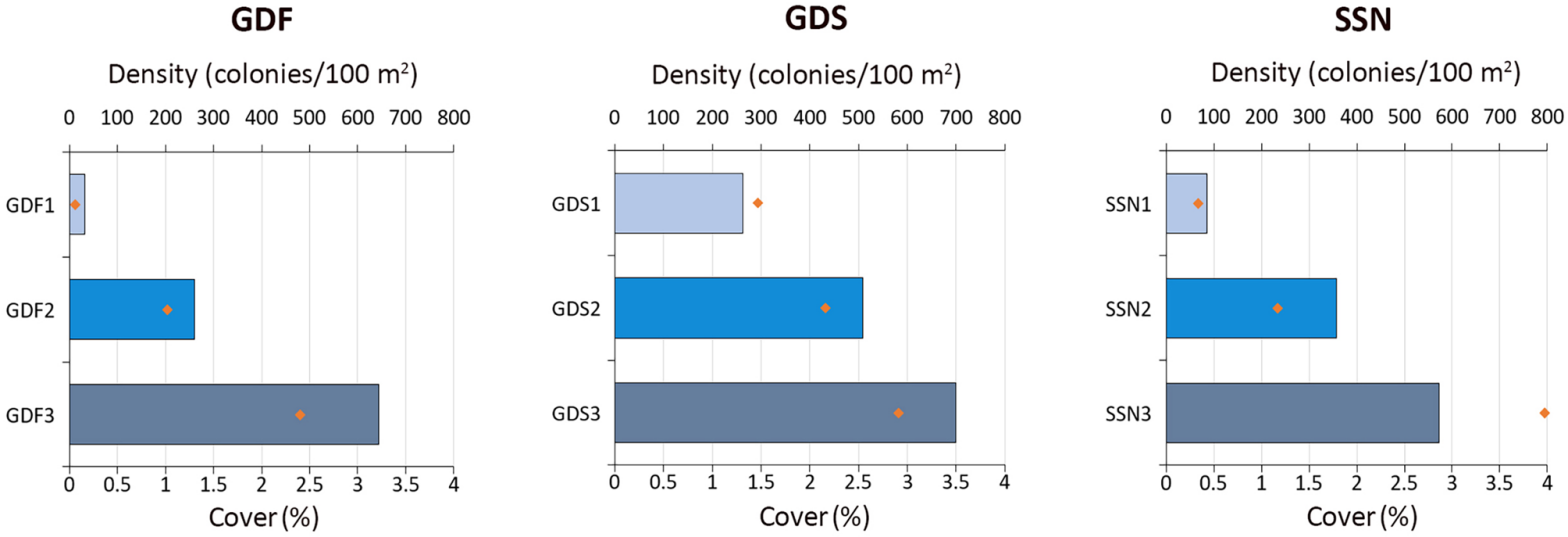
Density (bars) and cover (dots) of *Cladocora caespitosa* along three depth ranges (1: 14.5–17 m; 2: 17.1–19.5 m; 3: 19.6–22 m) at each site (GDF, GDS, SSN).

A total of 5133 *C. caespitosa* colonies were counted, photographed and measured along nine underwater transects on the corallith bed, with colony density increasing with depth (Table S1; Fig. 2). Density ranged between 31 and 263 colonies 100 m^− 2^ at 14.5–17.0 m depth, 260–509 colonies 100 m^− 2^ at 17.1–19.5 m depth, and 573–700 colonies 100 m^− 2^ at 19.6–22.0 m depth. Considering the total number of colonies within the three sites, 11% of them were found at 14.5–17 m depth (*n* = 568), 33% at 17.1–19.5 m depth (*n* = 1689), and 56% at 19.6–22.0 m depth (*n* = 2876). Due to increased colony density, distance within different colonies decreased with depth, with colonies living quite close to each other below 20 m depth, occasionally coalescing and aggregating in dense *C. caespitosa* beds. Even the largest colonies were detached from the seabed, although they did not have a round shape anymore. Similarly, the cover percentage increased with depth, with 0.1–1.5%, 1.0–2.2% and 2.4–4.0% at the three depth ranges, respectively.

### Biometry and population size structure

**Fig. 3:**
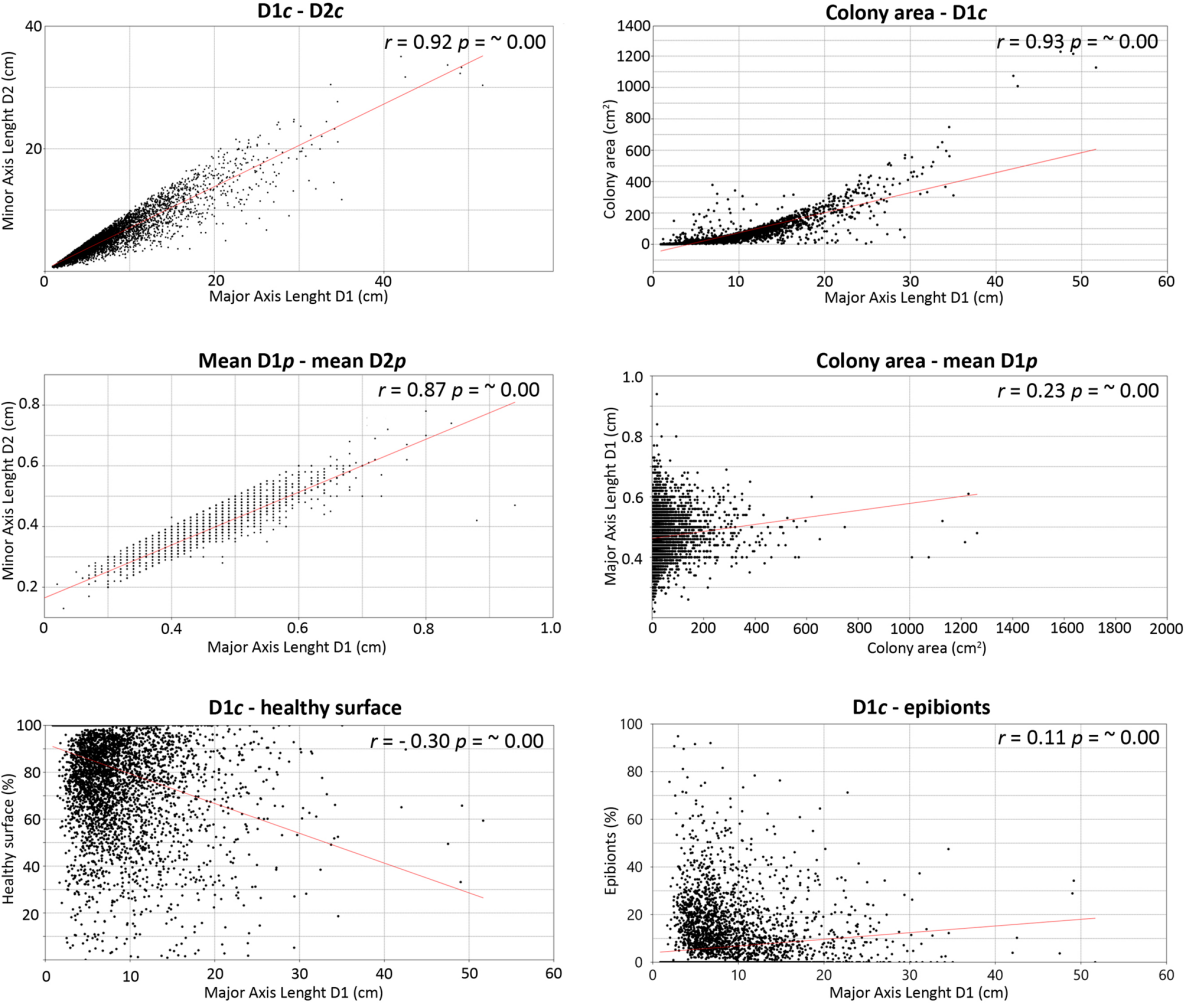
Spearman’s linear correlation among the biometric parameters and the conservation status measured on 5133 *Cladocora caespitosa* colonies; *r-* and *p-* values per each correlation are reported.

**Fig. 4:**
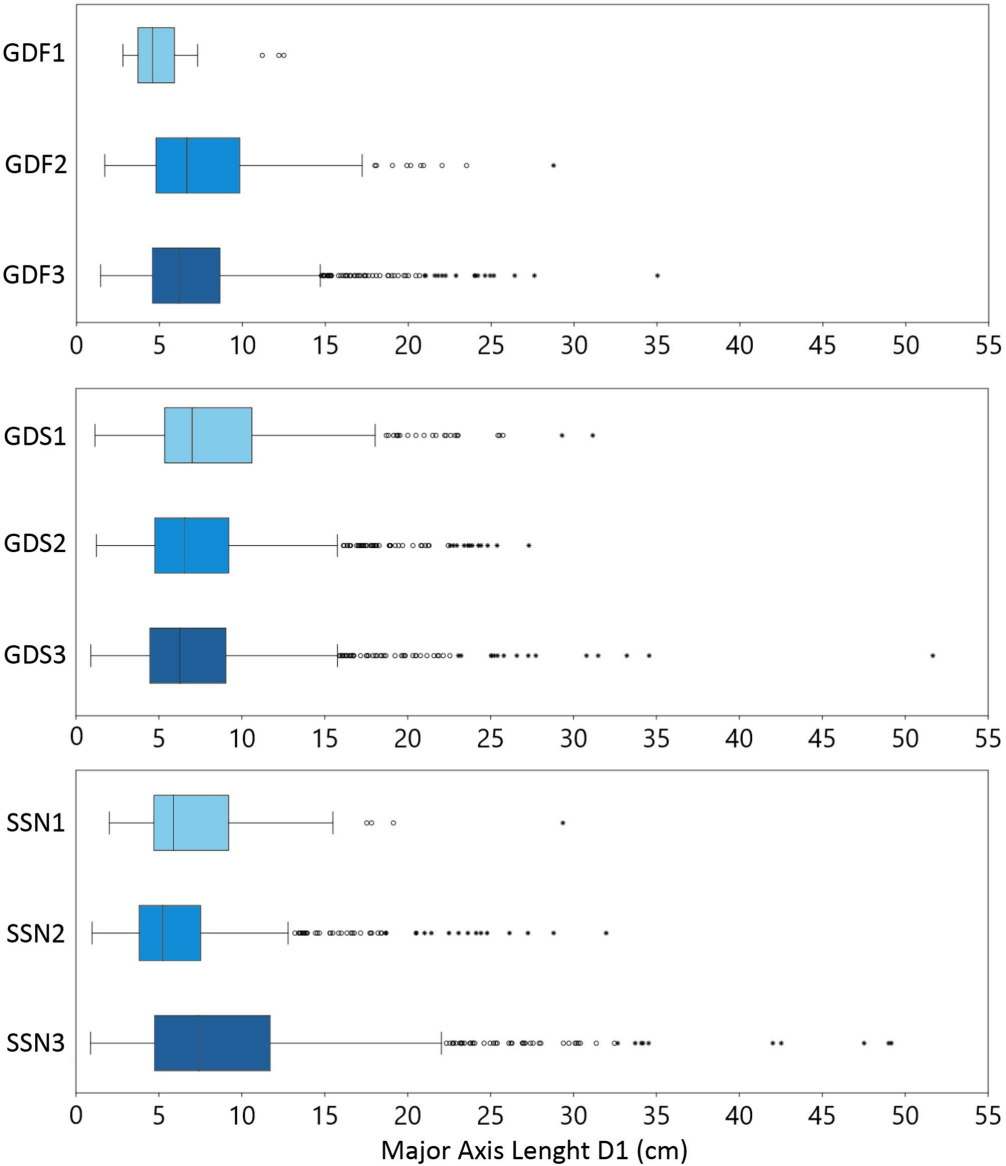
Boxplot of D1*c* (major axis) of *Cladocora caespitosa* showing median (vertical line), 25th and 75th percentiles (boxes), and minimum and maximum values smaller than 1.5 times the interquartile range (bars) at each transect; dots represent outliers.

A strong, positive correlation was found between colony major axis D1*c* and minor axis D2*c*, as well as between D1*c* and colony area (Fig. 3). Thus, D1*c* was selected as the main descriptor for colony size of *C. caespitosa*. Values of D1*c* ranged between 0.89 and 51.66 cm. Most of the population showed a D1*c* between 5 and 10 cm in all investigated transects and sites, while colonies larger than 15 cm D1*c* were generally present as outliers (Fig. 4). Largest colonies were broadly present in the deepest areas, although a general trend size/depth was not detected as it varied from site to site (Fig. 4; Table S2). For instance, within the three bathymetric ranges considered for each site, colonies were significantly larger in the shallowest area of GDS (14.5–17.0 m; GDS1), in the intermediate area of GDF (17.1–19.5 m; GDF2) and the deepest area of SSN (19.6–22 m; SSN3).

The majority of small (< 10 cm) and intermediate (10–20 cm) colonies showed spherical/elliptical shapes, while large colonies (> 20 cm) were irregular, with D1*c*–D2*c* and D1*c*–area correlations weakening with size (Fig. 3), the larger the colony the more irregular the shape.

**Fig. 5:**
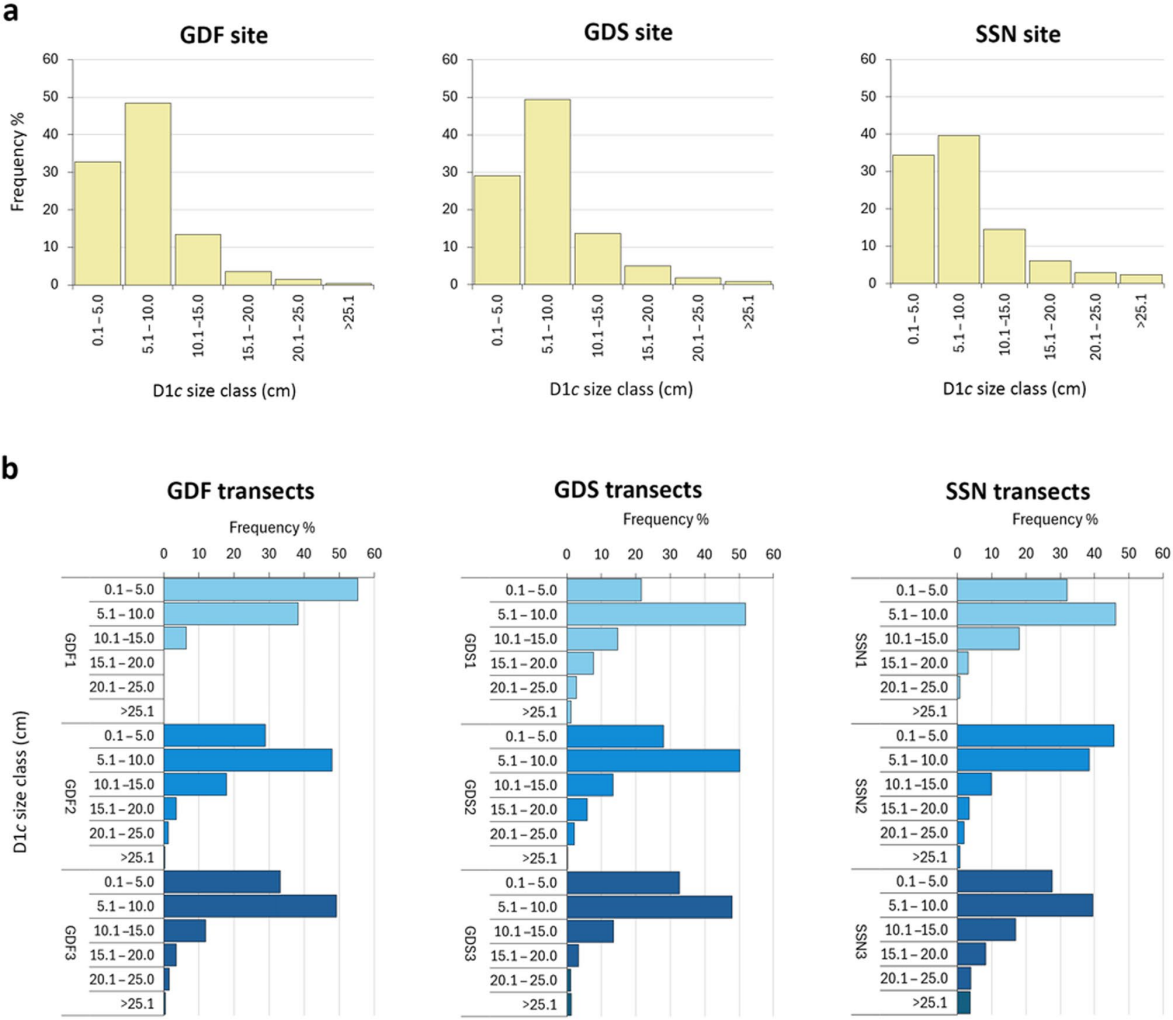
Size-frequency distribution of *Cladocora caespitosa* at Tremiti Islands (**a**) at each study site; (**b**) at each bathymetric range (14.5–17 m; 17.1–19.5 m; 19.6–22 m).

Size-frequency distribution based on D1*c* was unimodal and positively skewed at all study sites, with a right tail represented by colonies larger than 15 cm (Fig. 5). Skewness ranged from 1.40 to 2.50 among the different transects, indicating the prevalence of small classes (< 10 cm), which represented most of the population (70–95%).

Corallites showed a regular elliptical shape with mean values of D1*p* and D2*p* positively correlated, except for a few corallites larger than 8.5 mm (Fig. 3). D1*p* was an appropriate descriptor for corallites’ size as an indicator of polyps’ size, while no correlation was found between D1*p* and colony area (Fig. 3). Measured D1*p* ranged from 1.0 to 10.0 mm, with mean values between 3.5 ± 0.7 and 4.9 ± 0.9 mm. Both maximum and mean D1*p* broadly increased with depth at all study sites (Table S1).

### Conservation status

**Fig. 6:**
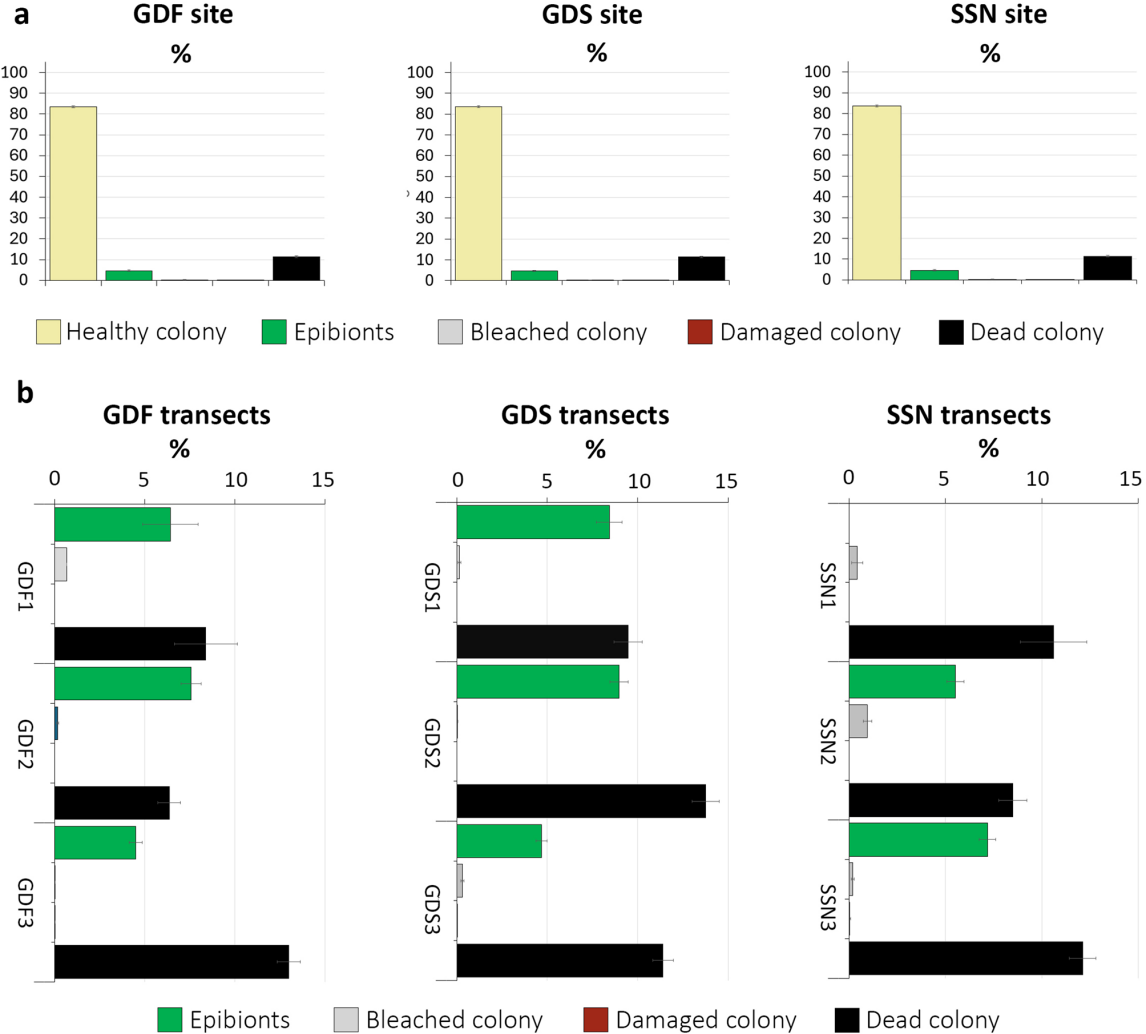
Conservation status of *Cladocora caespitosa*. Mean colony area percentage (± standard error) occupied by each category per (**a**) study site and (**b**) transect.

Mean colony area with living, healthy polyps ranged from 77.21 ± 0.86 to 88.97 ± 1.73% among the different transects, showing that colonies were in overall good conservation status at all study sites. A total of 1816 colonies (35.36% of the observed ones) were 100% healthy, with no signs of stress or impact. These colonies ranged from 0.9 to 35.0 cm D1*c*, while a negative correlation was found between D1*c* and the healthy surface (Fig. 3). Only three colonies (0.06% of all the colonies observed) were completely dead, while 2226 (43.35%) had some dead polyps, necrosis covering a mean colony surface between 8.39 ± 1.75 and 13.76 ± 0.74% among the transects (Fig. 6). The necrotic zones were often characterized by polyps parallel to the bottom, while living polyps were broadly perpendicular, suggesting that partial mortality might be also due to fragmentation as well as rollover and overturning events potentially caused by strong bottom currents and mechanical impacts.

Epibiosis characterized a total of 767 colonies, corresponding to 14.94% of the observed colonies in the whole study area, with epibionts covering between 0.14% and 65.33% of each colony. The mean epibiosis cover percentage per transect ranged from 4.50 ± 0.37% (GDF3) to 8.98 ± 0.50% (GDS2), while one transect (SSN1) showed no epibiosis on *C. caespitosa*. Epibionts were settled on dead portions of the colonies or within the interstices among living polyps. Epibiosis varied among the different sites without a clear pattern, decreasing with depth at GDF and GDS sites, while increasing at SSN. A slightly positive correlation was found between the epibionted surface and the size of the corals (Fig. 3). Epibiont composition was similar at all study sites, with the alien, invasive green algae *Caulerpa cylindracea*, the red algae *Wrangelia penicillata*, and the brown algae *Dyctiota linearis* being the most common species all over the investigated area. In particular, *C. cylindracea* formed dense turfs that patchy covered the seabed including colonies of *C. caespitosa*. Other observed epibionts include encrusting red algae in the genera *Lithophyllum* and *Peyssonnelia*, chlorophytes *Acetabularia acetabulum*, *Codium bursa*, *Halimeda tuna*, *Anadyomene stellata*, *Cladophora coelothrix*, *Pseudochlorodesmis furcellata*, and *Palmophyllum crassum*, brown algae *Dyctiota dichotoma* and *Padina pavonica*, as well as the sponge *Sarcotragus foetidous*, the cnidarian *Nemertesia antennina* and the bryozoan *Reteporella* sp.

Mechanical damage was observed in 0.07% of the colonies, which were partially broken. Damage was detected only at the deepest transects (GDS3, GDF3 and SSN3) where the mean damaged colony area varied from 0.01 ± 0.01 to 0.04 ± 0.03%. In many cases, fresh marks were present near damaged colonies, suggesting that this direct impact was likely caused by anchoring. Only recent damage was quantified since damaged colony areas tend to die and be colonized by epibionts.

Bleaching affected 2.18% of the colonies. The mean bleached area per colony varied from 0.01 ± 0.01 to 0.95 ± 0.22% within each transect. By considering only those colonies characterized by bleaching, it occupied from 0.18 to 64.14% of the corallum area. No colonies of *C. caespitosa* were completely bleached.

For each category, differences were observed among the transects, although without any specific geographic or bathymetric pattern (Table S2).

### Water column

**Fig. 7:**
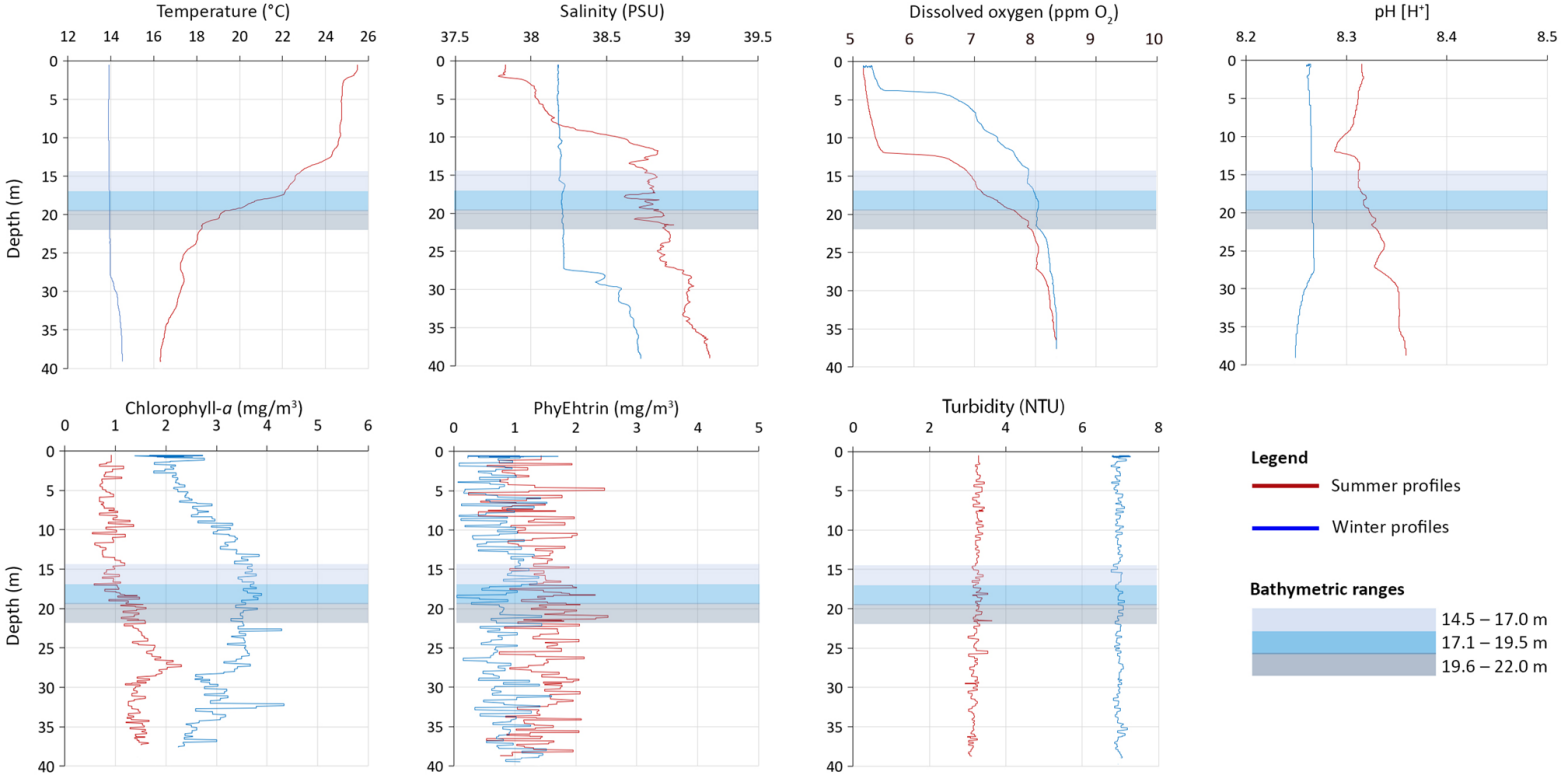
Mean physicochemical profiles recorded during winter and summer from the surface to 40 m depth in the study area. Coloured bars highlight the three bathymetric ranges with the *Cladocora caespitosa* bed considered in this study.

Temperature profiles highlighted the presence of a summer thermocline between 13 and 22 m depth, where the temperature dropped from about 25 °C to 18 °C. On the contrary, no thermocline was recorded in winter, when the water temperature was about 14 °C all along the water column up to ca. 30 m depth (Fig. 7). Water masses within the monitored *C. caespitosa* populations were broadly characterized by homogeneous salinity during winter (38.2–38.3 PSU) and by slightly more saline water lenses during summer (38.6–38.9 PSU). Dissolved oxygen increased with depth, particularly during summer. Seawater pH was slightly more acidic in summer (8.28–8.36) than in winter period (8.25–8.27) all along the depth gradient. Turbidity was constant along the water column, with higher - almost double - values in winter compared to summer, in accordance with the increased winter productivity highlighted by higher Chlorophyll-*a* concentration indicating higher phytoplankton density.

## Discussion

### Revising the nomenclature of *C. caespitosa* formations: beds, grounds and reefs

The term “corallith beds” has been recently proposed to describe an aggregation of coralliths made by the colonial scleractinian *Madracis decactis* off southern Brazil (tropical South Atlantic)^16^, and should be also adopted for *C. caespitosa*, *Cladocora* bed representing a dense population of numerous but spaced colonies on soft bottoms. On the contrary, *C. caespitosa* populations of numerous, spaced colonies attached to a rocky substrate^6,7^ should be called grounds, as already in use in comparable situations such as sponge grounds^37^. Banks are the most three-dimensional bioconstructions of *C. caespitosa*, built up by numerous large colonies often coalescing, and may deserve to be called reefs^10^. Two or more different formations can co-occur.

### A dense and peculiar Cladocora bed

The presence of an extensive bed of *C. caespitosa* entirely made by coralliths, on a soft bottom mostly at 17–22 m depth, is described here for the first time together with major ecological and biometric information. Coralliths of *C. caespitosa* have been previously reported only from one location (Espardelló islet, Formentera), at 5–13 m depth, among a majority of colonies attached to the substrate and within a *Cystoseira* association, with coralliths representing ca. 12% of the total colonies observed^11^. Other *C. caespitosa* grounds found so far are reported mainly in shallow rocky areas, often shallower than 10 m depth^8,9,11^. Their absence on soft bottoms is attributed to both instability and susceptibility to waves and currents, which can antagonize the settlement of larvae and the survival of small colonies, especially in exposed and/or highly hydrodynamic areas^38^. We observed the opposite situation at Tremiti Islands, since colonies were occasionally living on hard substrates at any depth, and rarely present shallower than 15 m depth on both rocky and soft bottoms, while the corallith bed was present on a pebbly, detritic seabed with rhodoliths. Both corallith and rhodolith beds, indeed, are considered *facies* of the Coastal Detrital biocoenosis (see^10,23^ and references therein), although rhodolith beds have been much more studied than corallith beds, in the Mediterranean as elsewhere. The occurrence of both coralliths and rhodoliths is also known from southern Brazil, where these two formations have been reported to form mixed beds together with bryoliths^16^.

As for rhodoliths, bottom currents are likely to shape the growth of *C. caespitosa* as coralliths at Tremiti Islands^20^. Similarly, coral fragments of both *Acropora anthocercis* from the Red Sea and *Porites lutea* from the Great Barrier Reef (Australia) - originating from either asexual fragmentation or larval recruitment - display a spherical growth as the result of periodical rolling induced by strong currents^15,39^. The wave-induced rollover may contribute to the formation of coralliths, as reported from the Eastern Caribbean Sea where at least ten species of scleractinian corals are known to form coralliths in swell-exposed sites^40^. In addition, coralliths growth forms may be enhanced by bioturbators; for example, the spherical growth of *M. decactis* coralliths is influenced by rotatory movements caused by the sand dollar *Clypeaster subdepressus* (Gray, 1825) off Southern Brazil^16^ and by fish species in the Gulf of Panama^14^.

The reason why *C. caespitosa* prefers soft, detritic bottoms rather than rocky bottoms is still unresolved, although it could be due to less competition for space and no shading from macroalgae during the vegetative season. In fact, rhodolith beds are scantily colonized by sessile species and offer space for *C. caespitosa*, which adapted surprisingly well to incoherent substrates with the presence of carbonatic detritus.

Coral density was remarkably high. Although no density data are available for comparison with other corallith beds worldwide, the mean colony density per site at Tremiti Islands was broadly higher than what has been reported for any *C. caespitosa* ground^9,38,41^.

### Smaller colonies with smaller polyps

A right-tailed, skewed distribution seems to be a common feature in *C. caespitosa* size-frequency distribution, as recently found in other *C. caespitosa* beds^38,41^. Based on the dominant presence of colonies smaller than 10 cm at Tremiti Islands (D1*c* size classes 5–10 cm and < 5 cm), this population resulted overall composed of smaller colonies than other populations, such as the ones in the Gulf of Trieste (dominance of D1*c* size classes 5–10 cm and 10–15 cm^38^), in the Columbretes Islands (dominance of D1*c* size classes 10–20 cm and 20–30 cm^9^) and in the Menorca Biosphere Reserve (dominance of D1*c* size class 10–20 cm^35^). Similarly to the Tremiti Islands, small colonies are frequent in *C. caespitosa* populations from Formentera^12^, Eastern Ligurian Sea^3,41^ and Kotor Bay, Montenegro^8^. Such abundance indicates high recruitment rates. If combined with the mortality of large colonies, the proliferation of juveniles can indicate an increase in reproductive rate occurring under pre-lethal conditions (hormesis), as suggested for tropical scleractinians^42^.

Mean D1*c* at Tremiti Islands (5.19–9.34 cm) resulted lower compared to those from the Gulf of Trieste (7.87–17.90 cm^38^), Ligurian Sea (12.78–13.00 cm^28,43^), and Columbretes Islands (31.48 cm^9^). Similarly to D1*c*, mean D1*p* at Tremiti Islands (3.49–4.85 mm) was smaller than those reported from the Gulf of Trieste (4.32–5.35 mm^38^) and southern Croatian waters (4.97–5.77 mm^7^). Differences in corallite diameter might be related to bottom current regimes, with larger calyx usually found in sites under stronger currents^7^ or less disturbed conditions. This aspect might also explain the slight increase of D1*p* with depth, as well as the lack of correlation between D1*p* and colony area. In fact, polyp size is related to trophic conditions, currents and other environmental factors, whereas colony size is related to age and, eventually, coalescence, as supported by both the D1*c*–D2*c* and the D1*c*–area correlations.

### Emerging environmental drivers keep Cladocora beds in deeper areas

The scarce occurrence of *C. caespitosa* in areas shallower than 15 m depth at Tremiti Islands could be due to the action of waves and storms, particularly strong during winter considering the exposed position of the archipelago in the Adriatic Sea. The finding of some overturned colonies mostly along the shallowest transects (14.5–17.0 m depth) may corroborate this hypothesis. However, the overlapping between the presence of a dense *C. caespitosa* bed and the shallowest summer thermocline suggests that seawater temperature is the main driver determining the bathymetric distribution of these formations. The *C. caespitosa* bed develops under the thermocline protection from high summer temperatures - which can occur from July to September - finding refuge in deeper areas compared to what is expected for the species. In the last ten years, the summer thermocline at Tremiti Islands has moved deeper due to warmer surface temperatures and marine heatwaves^44^, with the concomitant shift of this natural barrier from summer heat. The progressive sinking of the upper thermocline, from ca. 5 to 10 m depth in the 2014–2020 time frame, and at 13 m depth in 2023, may impede the presence of extensive populations of *C. caespitosa* above this bathymetric limit. Similarly, other Mediterranean benthic species have been recently recorded finding refuge at depth to avoid the increased temperature variability in shallow waters (e.g^45^. , and references therein). Developing deeper to escape excessively warm temperatures has also been observed in several shallow-water species in tropical reefs, which are finding protection from rapid ocean warming at moderate depths (e.g^46–48^).

The development of *C. caespitosa* beds in deeper areas might represent a climate change refugia for the species. At this depth, however, *C. caespitosa* may rely more on heterotrophy due to scarce light intensity and increased water turbidity in winter. Summer season might be challenging also considering the increased salinity, the decreased dissolved oxygen, the more acidic seawater pH, and the reduced Chlorophyll-*a* concentration suggesting less food availability.

### Healthy but threatened

The conservation status of *C. caespitosa* was overall good all over the investigated area at all depths. Although the *C. caespitosa* bed lays within the least protected portion of the MPA (Zone C), it likely benefits from the limitation of some local stressors (e.g., coastal development, pollution, high sedimentation rates, trawl fishing) thanks to the presence of the MPA. No recent mortality events were detected, nor were relevant cases of massive epibiosis, bleaching, or anthropogenic mechanical damages, except for a few impacts by boat anchors. Some necrosis might be related to rollover due to strong bottom currents rather than physiological stress, as also attested by the very low incidence of bleaching. The latter can be related to the depth of occurrence where seawater temperatures are always below 28 °C, which is the threshold temperature triggering bleaching and fast mortality in *C. caespitosa*^28^. This is supported by the upper limit of occurrence of the species at Tremiti Islands, mostly around 12 m depth, which is the shallowest portion of the seabed that does not experience prolonged (a few weeks) exposure to temperatures warmer than 24 °C that are considered stressful for *C. caespitosa*^28^.

Necrotic portions not directly attributable to rollover were mainly found on medium and large-sized colonies that, however, were still overall healthy. The low incidence of recent necrosis and epibiosis - the latter considered as an indicator of old necrosis due to any impact, rollover or bleaching - emphasizes the good conservation status of *C. caespitosa* below 15 m depth, while the rarity of this species at shallower depths suggest that summer conditions are too harsh for the survival of most of the colonies above this bathymetric threshold. Epibiosis by the invasive algae *C. cylindracea* was overall low and did not seem to impact on *C. caespitosa*, differently from what reported from the eastern coast of the Adriatic Sea where *C. cylindracea* reaches up to 100% cover, directly affecting the coral polyps^13^.

Considering the rarity of large biogenic Mediterranean coral formations such as *C. caespitosa* beds and banks, their conservation is to be taken into serious account in light of potential threats for this keystone species. Despite the current good conservation status at Tremiti Islands, *C. caespitosa* is undergoing both bleaching and mortality events in other Mediterranean areas^49^. The large number of juvenile colonies observed might be a warning of a possible stressed status of the populations, which perhaps can escape into deep refugia through massive recruitment. Long-term monitoring programs will unveil if this recruitment will lead to a consistent renewal of the population and at what depth, or if recurrent heatwaves will humper recruits survival and growth, affecting the population. For this reason, preventing other forms of local anthropogenic impacts is necessary to avoid further stress on the coral populations and enhance their resilience. For instance, a redefinition of the MPA boundaries and zonation is urgently needed at Tremiti Islands to include *C. caespitosa* corallith beds at least in the partially protected zone (Zone B). In addition to MPAs, a solution towards the conservation of *C. caespitosa* formations is the implementation of Natura 2000 sites in EU waters.

## Conclusions

Tremiti Islands MPA hosts a deep and dense *C. caespitosa* corallith bed, with distribution patterns and demographic traits that can be considered exceptional. Its bathymetric occurrence is primarily influenced by seawater temperature. Hydrodynamism is possibly shaping the coralliths morphology and the overall small colony size observed. Substrate availability does not represent a primary driver in *C. caespitosa* distribution, as both rocky and detritic bottoms are widely present in the area from the coast down to more than 50 m depth. Food availability may affect the occurrence of *C. caespitosa* beds, possibly linked to bottom currents supplying organic matter and plankton, although no data are available about the diet and trophism of *C. caespitosa* in the study area. Ad hoc monitoring plans coupled with appropriate image analysis are effective in collecting a large amount of information and should be regularly adopted. This is particularly true within the MPAs, which can play an important role in studying, monitoring, and preserving endangered *C. caespitosa* formations all over the Mediterranean Sea.

## Materials and methods

### Study area

**Fig. 8:**
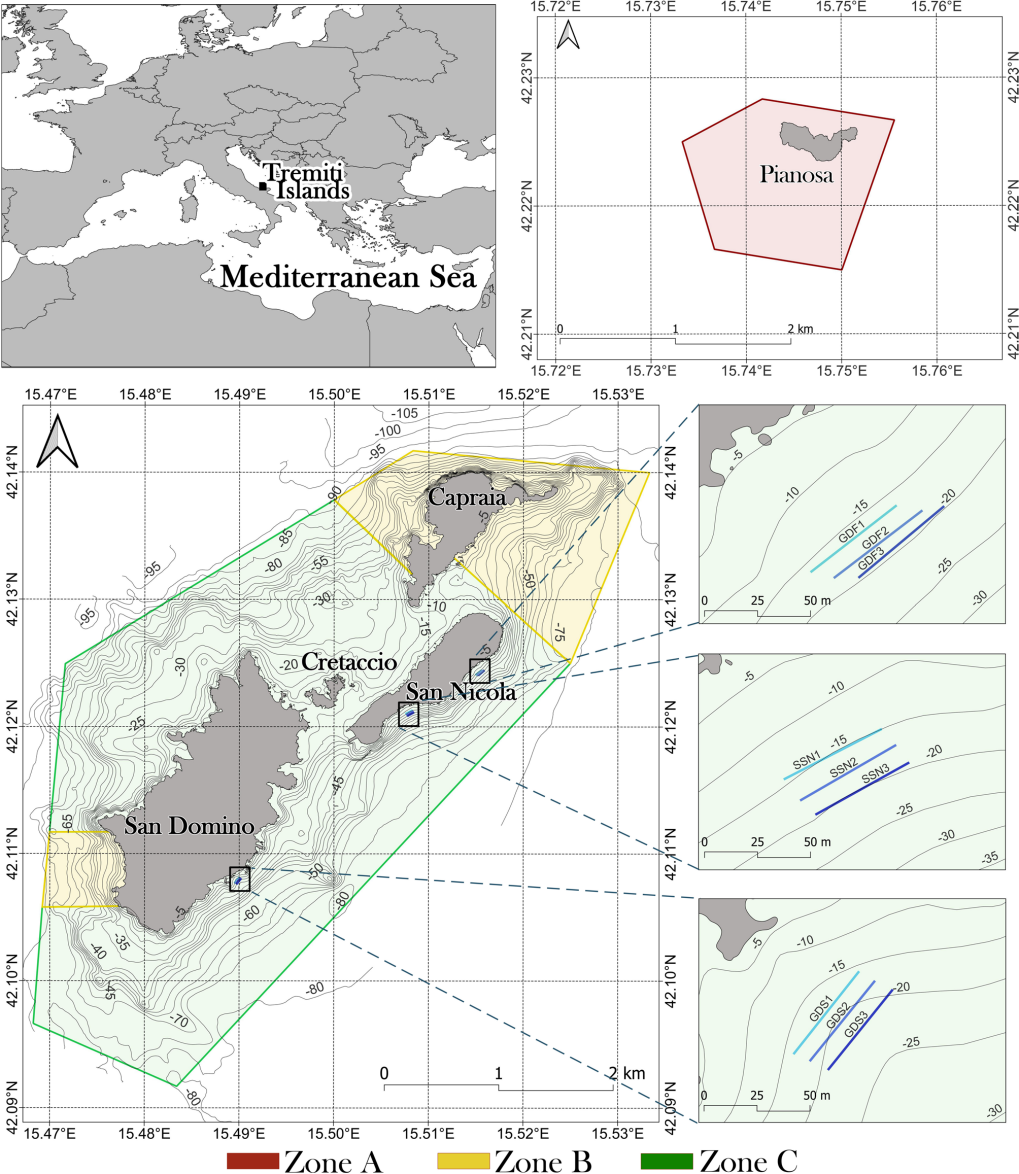
Map of the study area showing the three study sites with the indication of the three underwater transects carried out at each site. Marine Protected Area zonation: zone A, no take, no entry; zone B: partially protected; zone C: buffer. Isobaths: 5 m.

The study was carried out at Tremiti Islands MPA (42°07.38’N–15°30.02’E), located in the Southern Adriatic Sea, 12 nautical miles off the Gargano Promontory (Italy) (Fig. 8). The archipelago includes San Domino, San Nicola and Capraia Islands, a smaller rock called Cretaccio, as well as Pianosa Island located 11 nautical miles northeast of the three main islands. The MPA was established in 1989 and covers a maritime area of 1466 ha. It is organized into three main zones (Fig. 8): Zone A (no take, no entry zone; 178 ha), confined to Pianosa Island, where all human activities are forbidden; Zone B (partially protected zone; 261 ha) where anchoring and recreational fishing are forbidden, while scientific research, scuba diving, motor navigation and professional fishing are subjected to authorization; Zone C (buffer zone; 1027 ha), where scientific research and professional fishing have to be authorized, while any other activity (including anchoring and recreational fishing) is allowed.

The existence of some genetic exchange between *C. caespitosa* colonies from Tremiti Islands with other populations has been assessed^50^, while the presence and the extension of *C. caespitosa* ecosystems were unquantified in the archipelago.

### Data collection and analysis

The bottom areas characterized by the presence of *C. caespitosa* were chosen during preliminary underwater surveys all around the Tremiti archipelago, from 5 to 25 m depth. Three study sites - GDF (Grotta del Ferraio), SSN (Sud San Nicola) and GDS (Grotta del Sale) - were characterized by a dense presence of *C. caespitosa* and were investigated by SCUBA diving in July 2023. All three sites fall within the Zone C of the MPA. Three bathymetric ranges were considered at each study site: 14.5–17.0 m; 17.1–19.5 m; 19.6–22.0 m. Linear photographic transects parallel to the coastline were carried out at each bathymetric range, for a total of 3 photographic transects per study site and 9 photographic transects in the whole study area (Fig. 8; Table S3). Transects were allocated on gentle-sloped, detritic seabed with gravels and rhodoliths^20^, occasionally interspersed with rocks. Each transect, identified by two metal pickets connected through a guiding rope, covered a surface of 150 m^2^ (50 m in length × 3 m in width) (Fig. S2). Each colony of *C. caespitosa* present along the transect area was photographed from above using an Olympus TG-7 camera equipped with two laser beams for size reference (distance: 12 cm) and underwater lights. Each photo was analysed using photoQuad 1.4 software^51^ to measure different biometric parameters and to assess the conservation status of each colony. Biometry of each colony included the assessment of its surface area (cm^2^), as well as its major (D1*c*, cm) and minor (D2*c*, cm) axes. Furthermore, both major (D1*p*, mm) and minor (D2*p*, mm) axes of ten random corallites for each colony were measured. Both colony density (colonies 100 m^− 2^) and cover (percentage of the 150-m^2^ transect area occupied by *C. caespitosa* based on the surface of each colony) were assessed for each transect, as well as at site level (mean ± standard error). Mean colony D1*c*, D2*c*, D1*p* and D2*p* were also calculated for each transect and each site (mean ± standard error). Shapiro-Wilk test was run to verify the normality of data distribution using Past 4.03 software. Then, the correlation among the different biometric parameters was assessed through the non-parametric Spearman’s linear regression.

D1c was used to assess the population structure of *C. caespitosa* considering the following size classes: 0.1–5.0 cm; 5.1–10.0 cm; 10.1–15.0 cm; 15.1–20.0 cm; 20.1–25.0 cm; > 25.1 cm (*sensu*^38^). Size structure was analysed in terms of size frequency and distribution parameters, such as skewness and kurtosis.

The conservation status of each *C. caespitosa* colony was evaluated considering five main categories: healthy (polyps alive, typical brownish-green colour); bleached (polyps alive and transparent, colony resulting white); damaged (broken colony with exposed inner portion); dead (dead polyps); epibionted (presence of one or more organisms settled on the coral colony). Each category was quantified (cm^2^) and expressed as the percentage cover compared to the total area of each colony, then calculated at transect and site level (mean ± standard error).

The non-parametric Siegel-Tukey test was run to assess significant differences in both biometric and biological descriptors among bathymetric ranges.

Physicochemical parameters were measured during both winter and summer (March and July 2023) using a CTD Probe OS310 Idronaut. Temperature (°C), Salinity (PSU), Dissolved Oxygen (ppm O_2_), pH [H^+^], Chlorophyll-*a* (mg/m^3^), Phycoerythrin (mg/m^3^), and Turbidity (NTU) were recorded along the water column. Three profiles for each transect (*N* = 27) were recorded. Differences among data from different transects in the same season were tested using the non-parametric Siegel-Tukey test, then both winter and summer profiles were calculated considering the mean value of each parameter.

## Electronic supplementary material

Below is the link to the electronic supplementary material.

Supplementary Material 1

## Data Availability

The datasets generated during and/or analysed for the current study are available from the corresponding author upon request.

## References

[1] Kružić, P. & Požar-Domac, A. Skeleton growth rates of coral bank of *Cladocora caespitosa* (Anthozoa, Scleractinia) in lake Veliko Jezero (Mljet National Park). *Period Biol.* **104** (2), 123–130 (2002).

[2] Rodolfo-Metalpa, R., Peirano, A., Morri, C. & Bianchi, C. N. Coral calcification rates in the mediterranean scleractinian coral *Cladocora caespitosa* (L. 1767). *Atti Ass It Ocean. Limnol.* **13** (1), 291–299 (1999).

[3] Peirano, A., Morri, C. & Bianchi, C. N. Rodolfo–Metalpa, R. Biomass, carbonate standing stock and production of the mediterranean coral *Cladocora caespitosa* (L). *Facies* **44**, 75–80 (2001).

[4] Chefaoui, R. M., Casado-Amezúa, P. & Templado, J. Environmental drivers of distribution and reef development of the mediterranean coral *Cladocora caespitosa*. *Coral Reefs*. **36**, 1195–1209 (2017).

[5] Morri, C., Peirano, A., Bianchi, C. N. & Rodolfo-Metalpa, R. *Cladocora caespitosa*: A colonial zooxanthellate mediterranean coral showing constructional ability. *Reef. Encount*. **27**, 22–25 (2000).

[6] Peirano, A., Morri, C., Mastronuzzi, G. & Bianchi, C. N. The coral *Cladocora caespitosa* (Anthozoa, Scleractinia) as a bioherm builder in the mediterranean sea. *Mem. Descr. Carta Geol. D’It*. **52**, 59–74 (1998).

[7] Kružić, P. & Benković, L. Bioconstructional features of the coral *Cladocora caespitosa* (Anthozoa, Scleractinia) in the Adriatic sea (Croatia). *Mar. Ecol.* **29** (1), 125–139 (2008).

[8] Mačić, V., Đorđević, N. & Petović, S. First monitoring of *Cladocora caespitosa* (Anthozoa, Scleractinia) in the Boka Kotorska Bay (Montenegro). *Stud. Mar.* **32** (1), 26–32 (2019).

[9] Kersting, D. K. & Linares, C. *Cladocora caespitosa* bioconstructions in the columbretes Islands marine reserve (Spain, NW Mediterranean): Distribution, size structure and growth. *Mar. Ecol.* **33** (4), 427–436 (2012).

[10] Bianchi, C. N., Kersting, D. K., Kružić, P., Morri, C. & Peirano, A. Reefs of *Cladocora caespitosa* in Interpretation Manual of Marine Habitat Types in the Mediterranean Sea (eds Montefalcone, M., Bianchi, C. N., Bo, M. & Piazzi, L.) 131–134 (UNEP/MAP-SPA/RAC, 2021).

[11] Kersting, D. K., Cebrian, E., Verdura, J. & Ballesteros, E. Rolling corals in the mediterranean sea. *Coral Reefs*. **36**, 245–245 (2016).

[12] Kersting, D. K., Cebrian, E., Verdura, J. & Ballesteros, E. A new *Cladocora caespitosa* population with unique ecological traits. *Mediterr. Mar. Sci.* **18** (1), 38–42 (2017).

[13] Kružić, P. et al. A *Cladocora caespitosa* bank (National park Mljet, Adriatic Sea) under climate and anthropogenic impacts: A 20-year survey. *Mediterr. Mar. Sci.* **26** (1), 156–174 (2025).

[14] Glynn, P. W. Rolling stones amongst the Scleractinia: Mobile coralliths in the Gulf of Panama. In *Proc. 2nd Int. Coral Reef Symp.* 2,183–198 (1974).

[15] Roff, G. Corals on the move: Morphological and reproductive strategies of reef flat coralliths. *Coral Reefs*. **27**, 343–344 (2008).

[16] Capel, K. C. C., Segal, B., Bertuol, P. & Linder, A. Corallith beds at the edge of the tropical South Atlantic. *Coral Reefs*. **31**, 75 (2012).

[17] Hoeksema, B. W. & Wirtz, P. Over 130 years of survival by a small, isolated population of *Favia gravida* corals at ascension Island (South Atlantic). *Coral Reefs*. **32**, 551 (2013).

[18] Bosence, D. J. The occurrence and ecology of recent rhodoliths—a review in Coated Grains (ed Peryt, T. M.) 225–242 (Springer, 1983).

[19] Foster, M. S. Rhodoliths: Between rocks and soft places. *J. Phycol.* **37**, 659–667 (2001).

[20] Chimienti, G. et al. Rhodolith beds heterogeneity along the Apulian continental shelf (Mediterranean Sea). *J. Mar. Sci. Eng.* **8** (10), 813 (2020).

[21] James, D. W., Foster, M. S. & O’Sullivan, J. Bryoliths (Bryozoa) in the Gulf of California. *Pac. Sci.* **60** (1), 117–124 (2006).

[22] Klicpera, A., Taylor, P. D. & Westphal, H. Bryoliths constructed by bryozoans in symbiotic associations with hermit crabs in a tropical heterozoan carbonate system, Golfe D’Arguin, Mauritania. *Mar. Biodiv*. **43**, 429–444 (2013).

[23] Bianchi, C. N. & Morri, C. Different rhodolith assemblages host distinct associated species but similar ecological groups: A case study in NW mediterranean sea. *Aquat. Bot.* **196**, 103826 (2025).

[24] Aguirre, J. & Jiménez, A. P. Fossil analogues to present-day *Cladocora caespitosa* coral banks: Sedimentary setting, dwelling community, and taphonomy (Late pliocene, W Mediterranean). *Coral Reefs*. **17**, 203–213 (1998).

[25] Bernasconi, M. P., Corselli, C. & Carobene, L. A bank of the scleractinian coral *Cladocora caespitosa* in the pleistocene of the Crati Valley (Calabria, Southern Italy): Growth versus environmental conditions. *B Soc. Paleontol. Ital.* **36**, 53–62 (1997).

[26] Ingrosso, G. et al. Mediterranean bioconstructions along the Italian Coast. *Adv. Mar. Biol.* **79**, 61–136 (2018). 30012277 10.1016/bs.amb.2018.05.001

[27] Rodolfo-Metalpa, R., Bianchi, C. N., Peirano, A. & Morri, C. Coral mortality in NW mediterranean. *Coral Reefs*. **19** (1), 24 (2000).

[28] Rodolfo-Metalpa, R., Bianchi, C. N., Peirano, A. & Morri, C. Tissue necrosis and mortality of the temperate coral *Cladocora caespitosa*. *Ital. J. Zool.* **72** (4), 271–276 (2005).

[29] Kersting, D. K., Bensoussan, N. & Linares, C. Long-term responses of the endemic reef-builder *Cladocora caespitosa* to mediterranean warming. *PLoS ONE*. **8**, e70820 (2013). 23951016 10.1371/journal.pone.0070820 PMC3741371

[30] Jiménez, C. et al. Mortality of the scleractinian coral *Cladocora caespitosa* during a warming event in the Levantine sea (Cyprus). *Reg. Environ. Chang*. **16** (7), 1963–1973 (2014).

[31] Kružić, P., Lipej, L., Mavrič, B. & Rodić, P. Impact of bleaching on the coral *Cladocora caespitosa* in the Eastern Adriatic sea. *Mar. Ecol. Prog Ser.* **509**, 193–202 (2014).

[32] Kersting, D. K., Ballesteros, E., De Caralt, S. & Linares, C. Invasive macrophytes in a marine reserve (Columbretes Islands, NW Mediterranean): Spread dynamics and interactions with the endemic scleractinian coral *Cladocora caespitosa*. *Biol. Invasions*. **16**, 1599–1610 (2014).

[33] Kružić, P. & Požar-Domac, A. Impact of tuna farming on the banks of the coral *Cladocora caespitosa* in the Adriatic sea. *Coral Reefs*. **26**, 665–665 (2007).

[34] El Kateb, A., Stalder, C., Neururer, C., Pisapia, C. & Spezzaferri, S. Correlation between pollution and decline of scleractinian *Cladocora caespitosa* (Linnaeus, 1758) in the Gulf of Gabes. *Heliyon* **2**(11), e00195 (2016). 10.1016/j.heliyon.2016.e00195 PMC5121140 27896319

[35] Kersting, D. K., Cefalì, M. E., Movilla, J., Vergotti, M. J. & Linares, C. The endangered coral *Cladocora caespitosa* in the menorca biosphere reserve: Distribution, demographic traits and threats. *Ocean. Coast Manag*. **240**, 106626 (2023).

[36] de Casado, P. et al. *Cladocora caespitosa*. The IUCN red list of threatened species 2015, (2015). e.T133142A75872554.

[37] Enrichetti, F. et al. Keratose-dominated sponge grounds from temperate mesophotic ecosystems (NW mediterranean Sea). *Mar. Ecol.* **41**, e12620 (2020).

[38] Zunino, S. et al. The ecology of the mediterranean stony coral *Cladocora caespitosa* (Linnaeus, 1767) in the Gulf of Trieste (northern Adriatic Sea): A 30-year long story. *Mar. Biol. Res.* **14** (3), 307–320 (2018).

[39] Riegl, B., Piller, W. E. & Rasser, M. Rolling stones: First report of a free living *Acropora anthocercis* (Brook) from the red sea. *Coral Reefs*. **15**, 149–150 (1996).

[40] Hoeksema, B. W., Hassell, D., Meesters, E. H. W. G. & van Duyl, F. C. Wave-swept coralliths of Saba bank, Dutch Caribbean. *Mar. Biodiv*. **48**, 2003–2016 (2018). 10.1007/s12526-017-0712-5 PMC6404733 30931012

[41] Azzola, A. et al. Population structure change in a temperate reef coral after a quarter of century. *Estuar. Coast Shelf Sci.* **270**, 107851 (2022).

[42] Bianchi, C. N. et al. Le suivi du blanchissement Es Coraux aux Maldives: Leçons à tirer et nouvelles hypothèses. *Océanis* **29** (3–4), 325–354 (2003).

[43] Peirano, A., Morri, C. & Bianchi, C. N. Skeleton growth and density pattern of the temperate, zooxanthellate scleractinian *Cladocora caespitosa* from the Ligurian sea (NW Mediterranean). *Mar. Ecol. Prog Ser.* **185**, 195–201 (1999).

[44] Chimienti, G. et al. Effects of global warming on mediterranean coral forests. *Sci. Rep.* **11**, 20703 (2021). 34667231 10.1038/s41598-021-00162-4 PMC8526741

[45] Bianchi, C. N. et al. Consequences of the marine climate and ecosystem shift of the 1980-90s on the Ligurian sea biodiversity (NW Mediterranean). *Eur. Zool. J.* **86** (1), 458–487 (2019).

[46] Smith, T. B., Maté, J. L. & Gyory, J. Thermal refuges and refugia for stony corals in the eastern tropical pacific in *Coral Reefs of the Eastern Tropical Pacific; Coral Reefs of the World* (eds. Glynn, P., Manzello, D., Enochs, I.) 501–515 (Springer, 2015).

[47] Montefalcone, M., Morri, C. & Bianchi, C. N. Influence of local pressures on Maldivian coral reef resilience following repeated bleaching events, and recovery perspectives. *Front. Mar. Sci.* **7**, 587 (2020).

[48] Pancrazi, I., Ahmed, H., Chimienti, G. & Montefalcone, M. The Millepora zone is back: Recent findings from the northernmost region of the Maldives. *Diversity* **16** (4), 204 (2024).

[49] Garrabou, J. et al. Mass mortality in Northwestern mediterranean Rocky benthic communities: Effects of the 2003 heat wave. *Glob. Chang. Biol.* **15** (5), 1090–1103 (2009).

[50] López-Márquez, V. et al. Seascape genetics and connectivity modelling for an endangered mediterranean coral in the Northern ionian and Adriatic seas. *Landsc. Ecol.* **34**, 2649–2668 (2019).

[51] Trygonis, V. & Sini, M. PhotoQuad: A dedicated seabed image processing software, and a comparative error analysis of four photoquadrat methods. *J. Exp. Mar. Biol. Ecol.* **424**, 99–108 (2012).

