# Supplementary material for: Corallith bed of the endangered coral Cladocora caespitosa in the South Adriatic Sea
Source: Sci Rep. 2025 May 14;15:16690. doi: 10.1038/s41598-025-01554-6 (PMC12078607; doi:10.1038/s41598-025-01554-6)
Supplement: Supplementary file 1 — Supplementary Material 1 [file 41598_2025_1554_MOESM1_ESM.docx]

**Corallith bed of the endangered *Cladocora caespitosa* bed in the South Adriatic Sea**

Giovanni Chimienti^1,2^*, Andrea Tursi^1,2^, Alessia Logrieco^3^, Silvia Notarangelo^1^ and Francesco Mastrototaro^1,2^

^1^Department of Biosciences, Biotechnology, and Environment, University of Bari Aldo Moro, Via Orabona 4 70126, Italy

^2^CoNISMa, National Interuniversity Consortium for Marine Sciences, Piazzale Flaminio 9, 00197, Rome, Italy

^3^Department of Earth and geo-environmental sciences, University of Bari Aldo Moro, Via Orabona 4 70126, Italy

*Correspondence: giovanni.chimienti@uniba.it


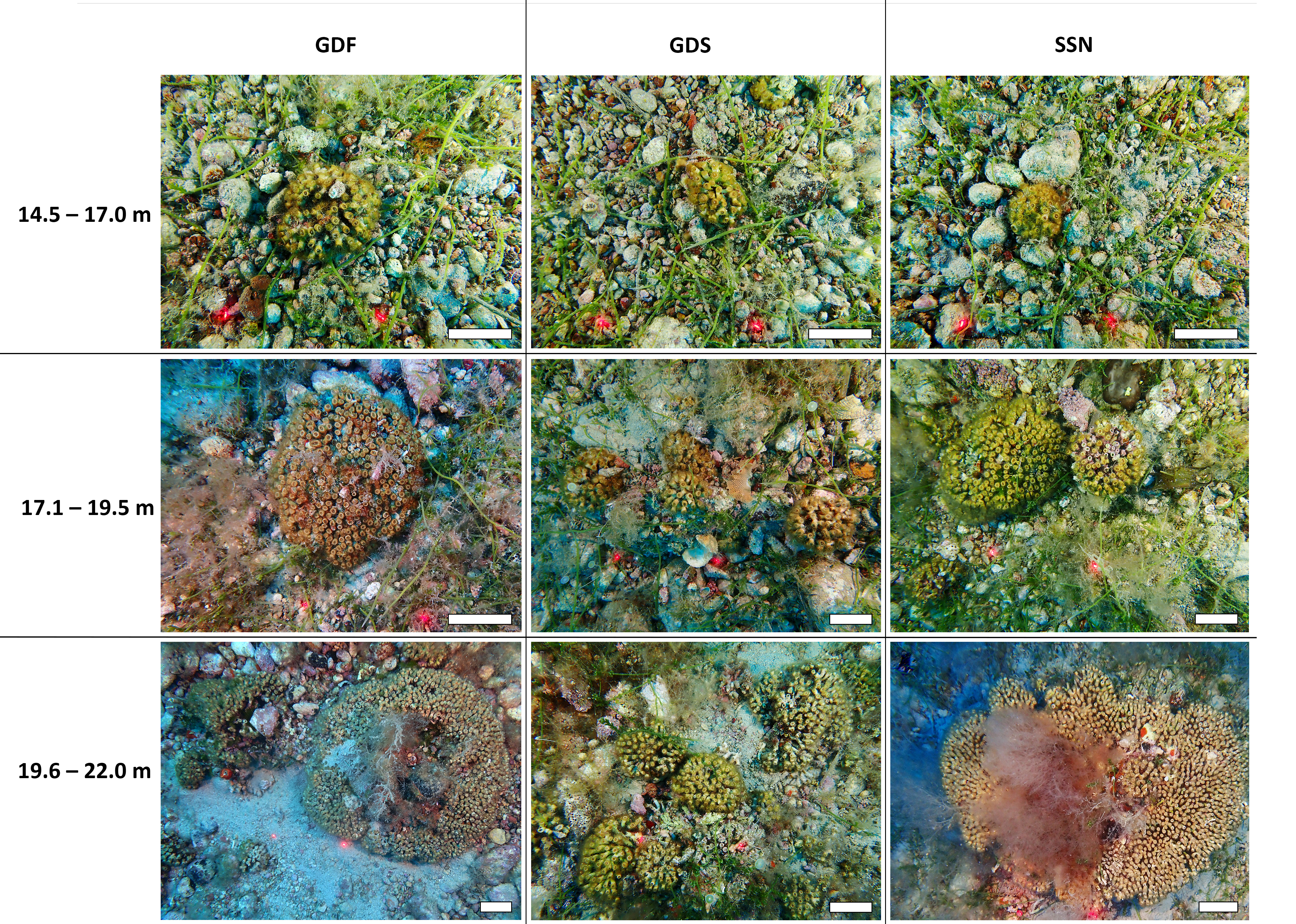


**Figure S1.** Example of *C. caespitosa* colonies at each study site (GDF, GDS, SSN) along the three bathymetric ranges investigated (14.5–17 m, 17.1–19.5 m, 19.6–22 m). Scale bar: 5 cm; lasers distance: 12 cm.

| Site | Transect | Depth (m) | N | Density  (col. 100 m^-2^) | Coverage  (%) | D1*c* (cm) | | | Skewness | Kurtosis | D1*p* (mm) | | |
| --- | --- | --- | --- | --- | --- | --- | --- | --- | --- | --- | --- | --- | --- |
|  |  |  |  |  |  | **Mean** | **Min.** | **Max.** |  |  | **Mean** | **Min** | **Max** |
| GDF | **GDF Site** | 14.5–22.0 | 1403 | 311.77±178.75 | 1.16±0.68 | 7.30±0.11 | 1.45 | 35.03 | 1.77 | 4.53 | 4.71±0.01 | 1.0 | 10.0 |
|  | **GDF1** | 14.5–17.0 | 47 | 31.33 | 0.06 | 5.19±0.31 | 2.80 | 12.50 | 2.05 | 4.75 | 3.49±0.03 | 2.0 | 6.0 |
|  | **GDF2** | 17.1–19.5 | 390 | 260 | 1.02 | 7.68±0.20 | 1.70 | 28.76 | 1.40 | 2.86 | 4.57±0.02 | 1.0 | 9.0 |
|  | **GDF3** | 19.6.–22.0 | 966 | 644 | 2.40 | 7.26±0.13 | 1.45 | 35.03 | 1.88 | 5.03 | 4.85±0.01 | 2.0 | 10.0 |
|  |  |  |  |  |  |  |  |  |  |  |  |  |  |
| GDS | **GDS Site** | 14.5–22.0 | 2206 | 490.66±126.59 | 2.18±0.42 | 7.69±0.10 | 0.89 | 51.66 | 1.98 | 6.88 | 4.70±0.00 | 2.0 | 9.0 |
|  | **GDS1** | 14.5–17.0 | 393 | 262.66 | 1.47 | 8.58±0.25 | 1.15 | 31.16 | 1.52 | 2.51 | 4.72±0.01 | 2.0 | 8.0 |
|  | **GDS2** | 17.1–19.5 | 763 | 509.33 | 2.16 | 7.66±0.16 | 1.23 | 27.31 | 1.52 | 2.48 | 4.64±0.01 | 2.0 | 8.0 |
|  | **GDS3** | 19.6.–22.0 | 1050 | 700 | 2.91 | 7.37±0.14 | 0.89 | 51.66 | 2.50 | 12.04 | 4.81±0.01 | 2.0 | 9.0 |
|  |  |  |  |  |  |  |  |  |  |  |  |  |  |
| SSN | **SSN Site** | 14.5–22.0 | 1524 | 338.66±141.18 | 1.82±1.10 | 8.18±0.15 | 0.88 | 49.16 | 2.22 | 7.22 | 4.64±0.01 | 2.0 | 9.0 |
|  | **SSN1** | 14.5–17.0 | 128 | 85.33 | 0.34 | 7.22±0.36 | 2.01 | 29.36 | 1.94 | 6.37 | 4.33±0.02 | 2.0 | 7.0 |
|  | **SSN2** | 17.1–19.5 | 536 | 357.33 | 1.17 | 6.55±0.20 | 0.97 | 31.97 | 2.20 | 6.02 | 4.49±0.01 | 2.0 | 8.0 |
|  | **SSN3** | 19.6.–22.0 | 860 | 573.33 | 3.97 | 9.34±0.23 | 0.88 | 49.16 | 2.01 | 5.83 | 4.82±0.01 | 3.0 | 9.0 |

**Table S1.** Depth, number of colonies (N), density, coverage, major colony axis (D1*c*), skewness, kurtosis, and major polyp’s corallite axis (D1*p*) at each study site and each transect. Mean values ± standard error are reported. Values at the site level have been obtained considering the total measurements of the three transects.

| STUDY SITE | GDF | | | GDS | | | SSN | | |
| --- | --- | --- | --- | --- | --- | --- | --- | --- | --- |
|  | **GDF1-2** | **GDF1-3** | **GDF2-3** | **GDS1-2** | **GDS1-3** | **GDS2-3** | **SSN1-2** | **SSN1-3** | **SSN2-3** |
| Colony area | 0.019* | 0.033* | 0.742 | 0.002* | 0.001*** | 0.961 | 0.763 | 0.004* | ~ 0*** |
| D1 colony | ~ 0*** | 0.002* | 0.194 | 0.005* | ~ 0*** | 0.395 | 0.479 | ~ 0*** | ~ 0*** |
| D1 polyps | ~ 0*** | ~ 0*** | ~ 0*** | 0.069 | 0.126 | ~ 0*** | 0.129 | ~ 0*** | ~ 0*** |
| Healthy colony | 0.389 | 0.541 | 0.873 | 0.001*** | 0.342 | ~ 0*** | 0.344 | ~ 0*** | ~ 0*** |
| Dead colony | 0.723 | 0.193 | ~ 0*** | 0.001*** | 0.195 | 0.022* | 0.465 | 0.698 | 0.001*** |
| Bleached colony | 0.001*** | ~ 0*** | 0.049* | 0.672 | 0.456 | 0.026* | 0.296 | 0.743 | ~ 0*** |
| Damaged colony | 1.000 | 0.967 | 0.785 | 1.000 | 0.711 | 0.591 | 1.000 | 0.813 | 0.536 |
| Epibionted colony | 0.807 | 0.446 | ~ 0*** | 0.751 | ~ 0*** | ~ 0*** | ~ 0*** | ~ 0*** | 0.018* |

**Table S2.** *p*-values of the non-parametric pairwise Siegel-Tukey test for significant differences among biometric and biological descriptors calculated for *Cladocora caespitosa* at Tremiti Islands Marine Protected Area. *p < 0.05; ***p < 0.001.

| Study Site | Transect | Depth range (m) | Start | | End | |
| --- | --- | --- | --- | --- | --- | --- |
|  |  |  | **Latitude N** | **Longitude E** | **Latitude N** | **Longitude E** |
| GDF | GDF1 | 14.5–17.0 | 42.124123 | 15.515048 | 42.124404 | 15.515539 |
|  | GDF2 | 17.1–19.5 | 42.124100 | 15.515182 | 42.124383 | 15.515686 |
|  | GDF3 | 19.6–22.0 | 42.124097 | 15.515319 | 42.124405 | 15.515812 |
| GDS | GDS1 | 14.5–17.0 | 42.107738 | 15.489584 | 42.108084 | 15.489955 |
|  | GDS2 | 17.1–19.5 | 42.107706 | 15.489679 | 42.108044 | 15.490049 |
|  | GDS3 | 19.6–22.0 | 42.107670 | 15.489779 | 42.108006 | 15.490146 |
| SSN | SSN1 | 14.5–17.0 | 42.121045 | 15.507654 | 42.121255 | 15.508214 |
|  | SSN2 | 17.1–19.5 | 42.120955 | 15.507752 | 42.121185 | 15.508293 |
|  | SSN3 | 19.6–22.0 | 42.120896 | 15.507838 | 42.121110 | 15.508365 |

**Table S3.** Coordinates and bathymetric ranges of the photographic transects carried out at each study site.


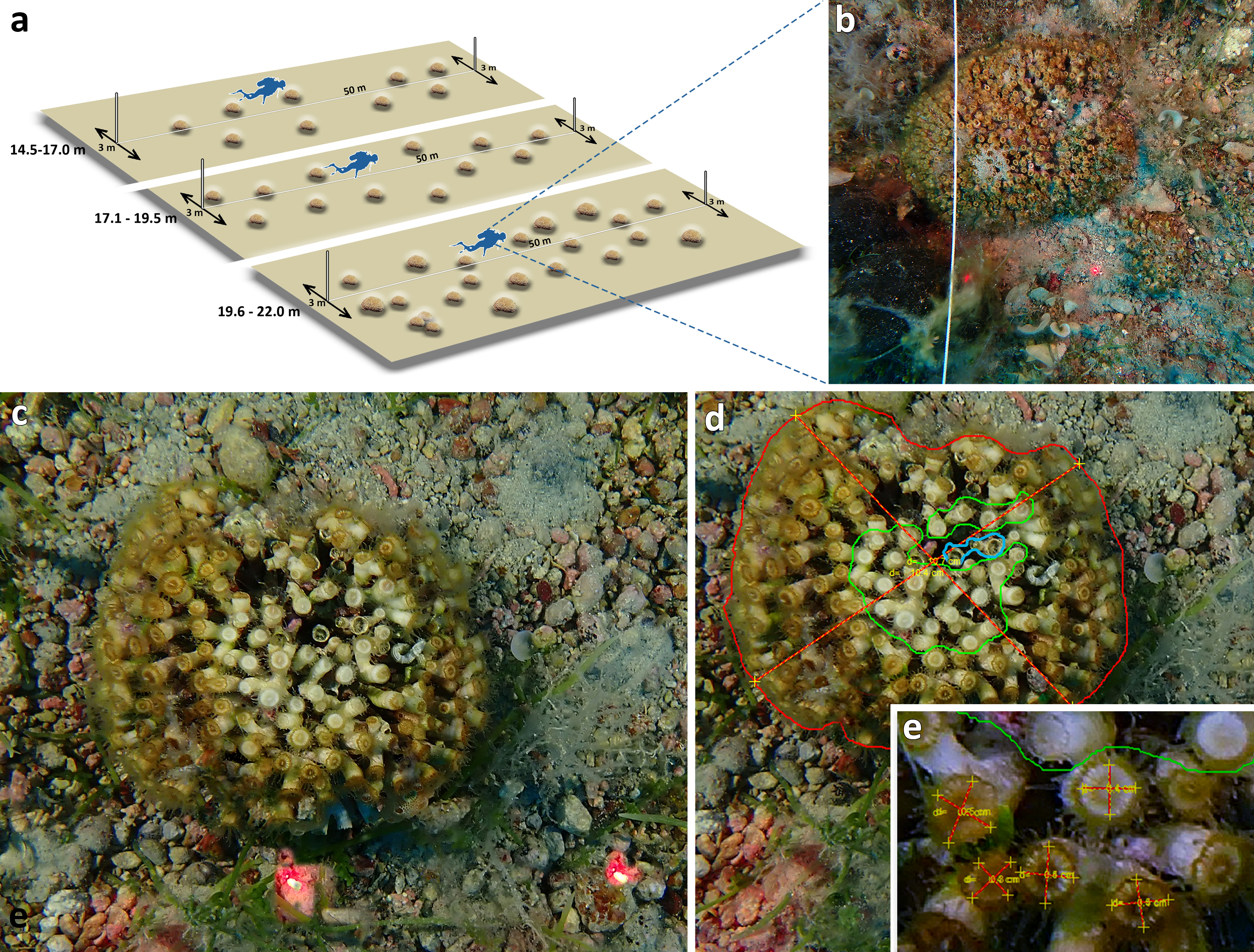


**Figure S2.** Image collection and analysis. a) Transects scheme with pickets, connected by a guiding rope, delimiting the transects area within the three bathymetric ranges; b–c) examples of photos taken for each colony; d) image analysis considering colony area (red), major and minor axis length (red lines), coverage of bleached polyps (green) and dead polyps (light blue); e) image analysis considering polyps major and minor axis (red lines). Laser: 12 cm.
